# Vasoactive intensity predicts mortality beyond SCAI staging in cardiogenic shock

**DOI:** 10.1093/ehjopen/oeag126

**Published:** 2026-08-18

**Authors:** Christophe Beyls, Nicolas Mollet, Nathan Helstroffer, Osama Abou-Arab, Yazine Mahjoub, Bélaïd Bouhemad, François Roubille, Pierre-Grégoire Guinot

**Affiliations:** Department of Anesthesiology and Intensive Care, Amiens University Hospital, 1 rond point christian Cabrol, AMIENS 80000, France; UR UPJV 7518 SSPC (Simplification of Care of Complex Surgical Patients) Research Unit, University of Picardie Jules Verne, 1 rond point christian Cabrol, AMIENS 80000, France; Department of Anesthesiology and Intensive Care, Amiens University Hospital, 1 rond point christian Cabrol, AMIENS 80000, France; Department of Anesthesiology and Intensive Care, Amiens University Hospital, 1 rond point christian Cabrol, AMIENS 80000, France; Department of Anesthesiology and Intensive Care, Amiens University Hospital, 1 rond point christian Cabrol, AMIENS 80000, France; Department of Anesthesiology and Intensive Care, Amiens University Hospital, 1 rond point christian Cabrol, AMIENS 80000, France; UR UPJV 7518 SSPC (Simplification of Care of Complex Surgical Patients) Research Unit, University of Picardie Jules Verne, 1 rond point christian Cabrol, AMIENS 80000, France; University of Burgundy and Franche-Comté, LNC UMR1231, 7 Bd Jeanne d'Arc, Dijon 21000, France; INSERM, LNC UMR1231, 7 Bd Jeanne d'Arc, Dijon 21000, France; FCS Bourgogne-Franche Comté, LipSTIC LabEx, 7 Bd Jeanne d'Arc, Dijon 21000, France; PhyMedExp, INSERM, CNRS, Cardiology Department, INI-CRT, University of Montpellier, 2 rue de l'Ecole de Médecine – 34000 MONTPELLIER, France; PhyMedExp, INSERM, CNRS, Cardiology Department, INI-CRT, University of Montpellier, 2 rue de l'Ecole de Médecine – 34000 MONTPELLIER, France; University of Burgundy and Franche-Comté, LNC UMR1231, 7 Bd Jeanne d'Arc, Dijon 21000, France; INSERM, LNC UMR1231, 7 Bd Jeanne d'Arc, Dijon 21000, France; FCS Bourgogne-Franche Comté, LipSTIC LabEx, 7 Bd Jeanne d'Arc, Dijon 21000, France; Department of Anesthesiology and Intensive Care, Dijon University Hospital, 1 Rue du Professeur Marion, Dijon F-21000, France

**Keywords:** Cardiogenic shock, VIS, SCAI staging, Vasopressin, Catecholamines

## Abstract

**Aims:**

Cardiogenic shock (CS) remains associated with high mortality. Severity is traditionally stratified by the Society for Cardiovascular Angiography and Interventions (SCAI) staging system despite substantial heterogeneity within stages. Whether vasoactive therapy intensity or combination provides additional prognostic information beyond SCAI staging remains unknown. This study assessed whether vasoactive support intensity, quantified by the vasoactive–inotropic score (VIS), improves mortality prediction beyond SCAI staging.

**Methods and results:**

In this retrospective bicentric cohort, consecutive adults admitted for CS between 2018 and 2023 were included. The VIS was calculated. The primary outcome was 90-day mortality. Multivariable logistic regression models adjusted for baseline severity assessed interactions with SCAI stage and the incremental prognostic value of VIS using nested models. Among 1167 patients, 90-day mortality increased across SCAI stages C, D, and E (20.1%, 27.4%, and 45.6%; *P* < 0.001, respectively), with substantial heterogeneity within stages. In stage C, dobutamine alone was associated with lower mortality than norepinephrine alone [OR = 0.25 (0.09–0.74); *P* = 0.009]; in stage E, dobutamine plus norepinephrine was associated with higher mortality [OR = 2.39 (1.07–5.36); *P* = 0.028]. The number of vasoactive agents was associated with mortality (OR = 1.27 per additional agent; *P* = 0.041) but proved a crude marker, providing only marginal incremental discrimination (*R*^2^ = 0.233; Δ = +0.011), with its prognostic information fully captured by VIS (*R*^2^ = 0.286 vs. 0.287 with and without number of agents). Adding SCAI stage minimally improved discrimination (*R*^2^ = 0.222–0.226; Δ = +0.004), whereas VIS substantially improved model performance (*R*^2^ = 0.286; Δ = +0.060; *P* < 0.001).

**Conclusion:**

Vasoactive intensity quantified by the VIS improved 90-day mortality prediction beyond SCAI staging and the simple count of vasoactive drugs, providing incremental prognostic information beyond SCAI staging.

## Graphical Abstract

**Graphical Abstract:**
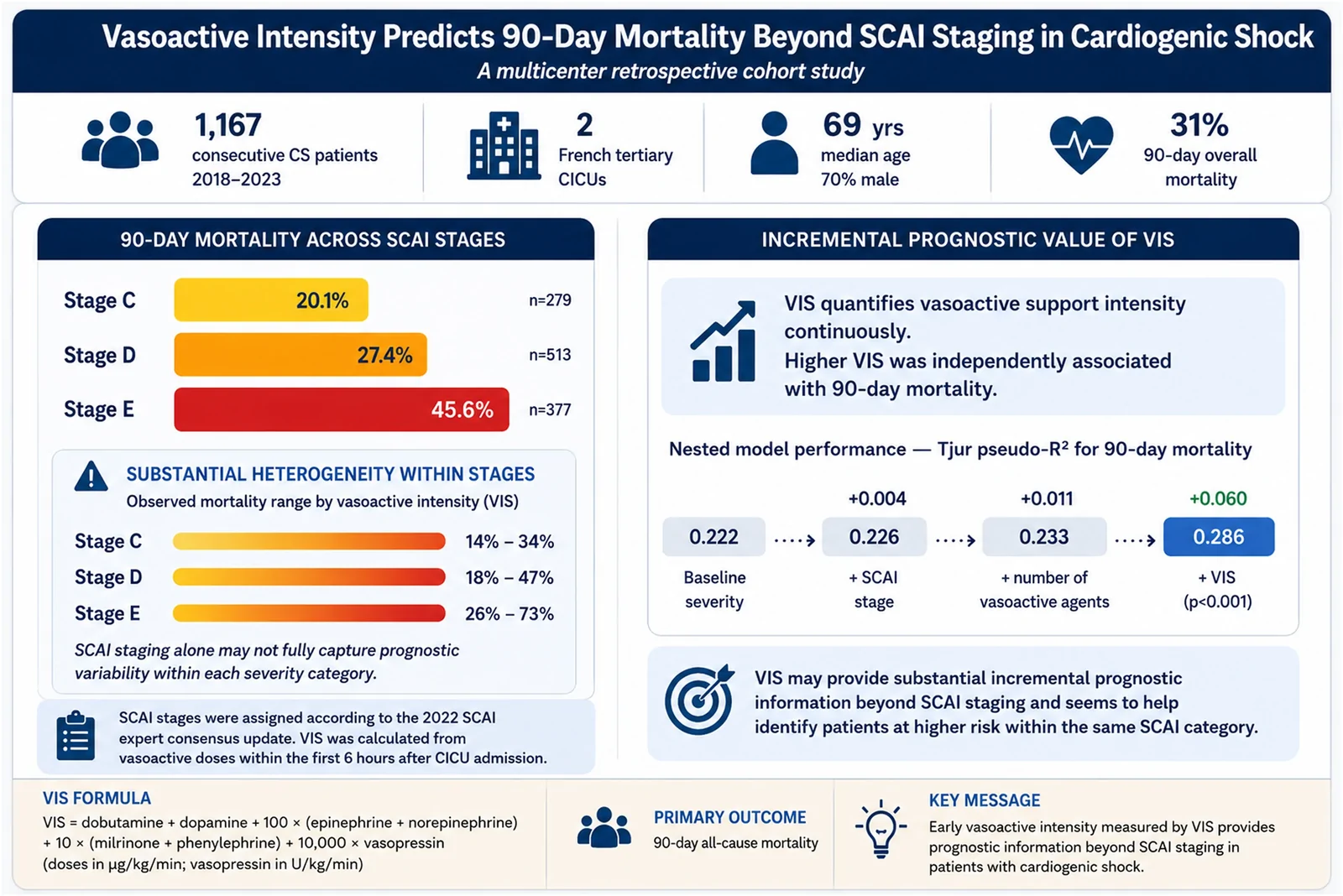

## Introduction

Cardiogenic shock (CS) is a severe form of circulatory failure associated with high mortality.^1^ The Society for Cardiovascular Angiography and Interventions (SCAI) classification has been proposed to standardize the assessment of shock severity and guide clinical management.^2^ This staging system integrates clinical signs of hypoperfusion, biomarkers, and the need for pharmacological or mechanical circulatory support. Several studies have confirmed a stepwise increase in mortality from stage C to stages D and E.^3–5^

Nevertheless, patients classified within the same SCAI stage may still represent a heterogeneous population with distinct clinical trajectories.^6^ While the SCAI classification captures key elements of shock severity, it may not fully account for differences in underlying haemodynamic profiles or in the intensity of circulatory support required during the early phase of shock.

In particular, the use of vasoactive agents varies widely in clinical practice. Some patients are managed with a single inotrope or vasopressor, whereas others require combinations of multiple agents to maintain adequate perfusion regarding the aetiology of CS.^6,7^ Such treatment patterns likely reflect varying degrees of circulatory failure and may therefore identify subgroups of patients with different prognoses even within the same SCAI stage.^8^

Current CS guidance relies on SCAI staging for risk stratification but does not incorporate a standardized measure of vasoactive support intensity.^9^ We therefore assessed mortality heterogeneity across vasoactive strategies within SCAI stages and evaluated the incremental prognostic value of the vasoactive–inotropic score (VIS) beyond staging, hypothesizing that vasoactive intensity captures prognostic information not reflected by SCAI classification alone.

## Materials and methods

PROMIX is a retrospective bicentric cohort study including all consecutive adults (≥18 years) admitted for cardiogenic shock to the cardiac intensive care units (CICUs) of two French academic hospitals (Amiens and Dijon) between January 2018 and December 2023.

Cardiogenic shock was defined and staged according to the 2022 SCAI expert consensus update.^10^ SCAI stage was prospectively recorded at CICU admission for patients included from 2021 onward, representing approximately two-thirds of the cohort. To ensure consistency across the entire study period, all patients were subsequently re-adjudicated according to the 2022 SCAI criteria using admission clinical, biological, and haemodynamic data. For patients enrolled before 2021, SCAI stage was assigned retrospectively using the same criteria.

Patients with SCAI stages C, D, or E were included. Eligible aetiologies included acute myocardial infarction, acute decompensation of chronic heart disease, arrhythmias, myocarditis, septic cardiomyopathy, and post-cardiotomy shock. Patients were excluded if shock was reclassified as non-cardiogenic or related to pulmonary embolism, toxic causes, peripartum cardiomyopathy, or extracorporeal cardiopulmonary resuscitation.

Vasoactive intensity was quantified using the VIS, calculated from the highest vasoactive doses recorded within the first 6 h after CICU admission^11^: VIS = dobutamine + dopamine + 100 × (epinephrine + norepinephrine) + 10 × (milrinone + phenylephrine) + 10 000 × vasopressin (units kg^−1^ min^−1^).

The primary outcome was 90-day all-cause mortality.

Multivariable logistic regression models adjusted for age, admission lactate, SOFA score, ECMO support, and post-cardiotomy status were used to assess the association between vasoactive support and mortality. As a sensitivity analysis, the final multivariable model was repeated separately in patients with prospectively assigned and retrospectively assigned SCAI stages to evaluate the consistency of the association between VIS and 90-day mortality.

Missing data were handled using multiple imputation. Three complementary dimensions were investigated: SCAI stage as a categorical severity indicator; vasoactive strategy, defined as the specific drug combination at baseline, evaluated through interaction terms with SCAI stage and within-stage contrasts using the Dunnett method; and vasoactive intensity, modelled both categorically (1, 2, or ≥3 agents) and continuously using VIS.

To assess whether the number of agents contributed information independently of VIS, which represents the weighted sum of vasoactive doses, both variables were entered simultaneously into a separate model.

Incremental prognostic value was assessed using nested models with likelihood-ratio testing and Tjur’s pseudo-*R*^2^. Model robustness was evaluated through calibration, discrimination, and multicollinearity.

A two-sided *P* < 0.05 was considered statistically significant. Analyses were performed using R software.

## Results

Among 1167 patients, 279 (24%) were classified as SCAI stage C, 513 (44%) as stage D, and 377 (32%) as stage E (*Table 1*). Ninety-day mortality increased stepwise across stages (20.1%, 27.3%, and 45.6%; *P* < 0.001), paralleled by progressive increases in lactate, SOFA score, and vasoactive support intensity.

**Table 1:** Baseline characteristics of patients according to SCAI shock stage at inclusion

	Stage C	Stage D	Stage E	*P* overall
	*N* = 279	*N* = 513	*N* = 377	
Age (years)	71.0 [64.0; 77.0]	68.6 [61.0; 75.0]	66.5 [58.0; 73.5]	<0.001
Male gender (*n*; %)	194 (69.5)	362 (70.6)	260 (69.0)	0.871
BMI (kg m^−2^)	25.95 [23.08; 30.32]	26.62 [23.67; 30.11]	26.22 [23.39; 29.73]	0.432
SAPS II	42.0 [32.5; 53.0]	47.0 [36.0; 65.0]	57.0 [42.0; 74.0]	<0.001
SOFA score	7.0 [5.0; 9.0]	8.0 [6.0; 11.0]	9.0 [7.0; 12.0]	<0.001
**SHARC Classification, *n* (%)**				
AMI-CS	31 (11.1)	92 (17.9)	143 (37.9)	<0.001
Stenting of culprit lesion	21 (7.53)	49 (9.55)	51 (13.53)	0.033
Post-cardiotomy	169 (60.6)	311 (60.6)	135 (35.8)	<0.001
Heart failure	4 (1.5)	49 (9.5)	22 (5.8)	<0.001
Secondary CS	75 (26.8)	61 (12)	77 (20.4)	<0.001
Cardiac arrest before inclusion	2 (0.72)	40 (7.80)	71 (18.83)	<0.001
Mechanical ventilation	198 (71.0)	394 (76.8)	268 (71.1)	0.085
**Number of vasoactive agent, *n* (%)**				
1	140 (50.18)	106 (20.74)	138 (36.60)	<0.001
2	139 (49.82)	337 (65.95)	174 (46.15)	
≥3	0 (0.00)	68 (13.31)	65 (17.24)	
**Vasoactive intensity at inclusion**				
VIS	19.00 [7.43; 52.00]	47.00 [20.50; 125.00]	94.52 [25.93; 212.01]	<0.001
VA-ECMO at time of VIS assessment	—	—	50 (13.2)	<0.001
**Biologic data at inclusion**				
pH	7.36 [7.31; 7.41]	7.33 [7.28; 7.39]	7.28 [7.18; 7.38]	<0.001
Lactate, (mmol l^−1^)	2.00 [1.50; 2.80]	2.80 [1.70; 4.80]	5.10 [2.30; 10.00]	<0.001
Serum creatinine, (µmol l^−1^)	91.0 [68.0; 121.5]	106.0 [78.0; 157.0]	111.0 [80.0; 171.0]	<0.001
**Clinical course, *n* (%)**				
VA-ECMO	14 (5.0)	142 (27.7)	163 (43.2)	<0.001
Renal replacement therapy	38 (13.62)	142 (27.68)	141 (37.40)	<0.001
90-day mortality	56 (20.07)	140 (27.29)	172 (45.62)	<0.001

Data are expressed as number (%), or median [interquartile range].

AMI, acute myocardial infarction; CS, cardiogenic shock; SAPS, simplified acute physiology score II; SOFA, sepsis organ failure assessment; VA-ECMO, veno-arterial extracorporeal membrane oxygenation; VIS, vaso-inotropic score.

Vasoactive strategies varied markedly across stages (*P* < 0.001): single-agent therapy predominated in stage C, two-drug combinations in stage D, and ≥3-agent regimens were mainly restricted to stages D and E, with no significant difference between these two stages after Bonferroni correction.

**Figure 1:**
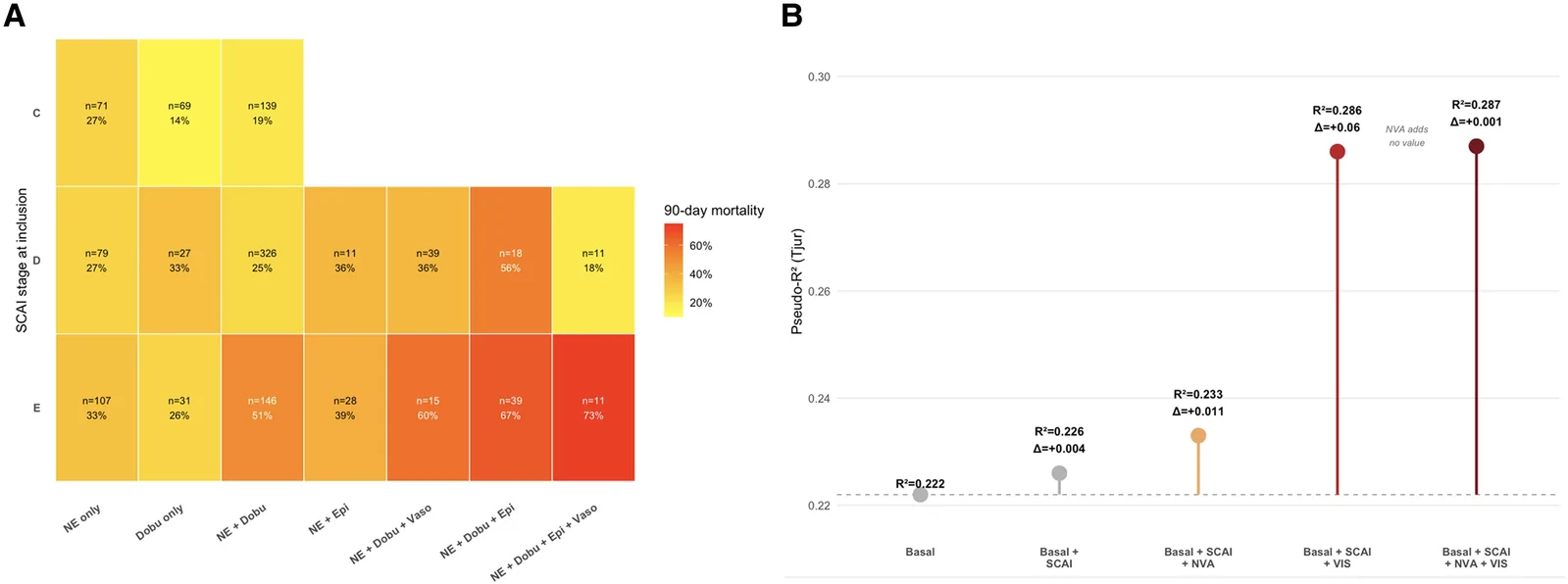
Mortality heterogeneity across vasoactive strategies within SCAI stages and incremental prognostic value of vasoactive intensity. (*A*) Heatmap of 90-day mortality according to vasoactive strategy within each SCAI stage at CICU admission. Each cell displays the number of patients and observed mortality rate. Cell shading reflects 90-day mortality, with increasing intensity indicating higher mortality. Cells with fewer than 10 patients are visually distinguished. Substantial heterogeneity in mortality is observed within each stage, particularly in SCAI stage E, where mortality ranges from 26% to 73% across strategies. (*B*) Incremental prognostic value for 90-day mortality. Pseudo-*R*^2^ (Tjur) from nested logistic regression models. Adding SCAI stage or number of vasoactive agents (NVA) to baseline severity provided minimal discrimination gain (Δ = +0.004 and Δ = +0.011), whereas VIS substantially improved model performance (Δ = +0.060; *P* < 0.001). Adding NVA to a model already containing VIS yielded negligible additional value (Δ = +0.001), indicating that VIS fully captures the prognostic information conveyed by the number of agents. Dobu, dobutamine; Epi, epinephrine; NE, norepinephrine; NVA, number of vasoactive agents; SCAI, Society for Cardiovascular Angiography and Interventions; Vaso, vasopressin analogue; VIS, vasoactive–inotropic score.

Within each SCAI stage, 90-day mortality varied substantially across vasoactive strategies (*Figure 1A*). In SCAI stage C, mortality ranged from 14% with dobutamine alone to 27% with norepinephrine alone. In SCAI stage E, this spread was even wider, ranging from 26% to 73%. In adjusted analyses (*Table 2*), dobutamine alone was associated with markedly lower mortality than norepinephrine alone in stage C [OR = 0.25 (0.09–0.74); *P* = 0.009]—a difference that disappeared in stages D and E [OR = 0.94 (0.25–3.61); *P* = 1.000]. Conversely, dobutamine plus norepinephrine was associated with higher mortality than norepinephrine alone specifically in stage E [OR = 2.39 (1.07–5.36); *P* = 0.028], but not in stages C or D. These stage-specific contrasts, though not supported by a significant overall interaction term (*P* = 0.43)—likely reflecting limited power in small subgroups—suggest that the prognostic impact of vasoactive combinations differs across the severity spectrum.

**Table 2:** Adjusted odds ratios for 90-day mortality by catecholamine strategy within each SCAI stage

Strategy (vs. NE only)	SCAI C	SCAI D	SCAI E
Dobu	0.25 (0.09–0.74); *P* = 0.009*	1.07 (0.27–4.20); *P* = 0.999	0.94 (0.25–3.61); *P* = 1.000
Dobu + NE	0.64 (0.27–1.49); *P* = 0.400	1.47 (0.64–3.37); *P* = 0.659	2.39 (1.07–5.36); *P* = 0.028*
NE + Epi	Ne	2.72 (0.38–19.49); *P* = 0.582	0.69 (0.19–2.55); *P* = 0.903
Dobu + NE + Epi	Ne	3.90 (0.78–19.52); *P* = 0.133	3.25 (0.97–10.86); *P* = 0.058
Dobu + NE + Vaso	Ne	1.23 (0.36–4.14); *P* = 0.977	1.38 (0.24–8.04); *P* = 0.971
Dobu + NE + Epi + Vaso	Ne	0.24 (0.02–2.54); *P* = 0.426	1.65 (0.23–12.00); *P* = 0.927

Odds ratios (95% CI) adjusted for age, lactate, SOFA, ECMO, and post-cardiotomy status.

*P*-values adjusted using the Dunnett method.

Dobu, dobutamine; Epi, epinephrine; NE, norepinephrine; Ne, not estimable; SCAI, Society for Cardiovascular Angiography and Interventions; Vaso, vasopressin analogue.

Vasoactive intensity was independently associated with 90-day mortality across all metrics. When modelled categorically, each additional vasoactive agent was associated with increased odds of death (OR = 1.27; *P* = 0.041). However, the number of agents proved a crude marker of support intensity: when VIS and number of agents were entered simultaneously, the number of agents lost prognostic significance, whereas VIS remained independently associated with mortality, and adding the number of agents to a model already containing VIS yielded negligible incremental discrimination (Tjur *R*^2^ = 0.287 vs. 0.286; Δ = +0.001, *Figure 1B*), indicating that VIS captures the full prognostic information conveyed by the number of agents.

In nested models (*Figure 1B*), adding SCAI stage to baseline severity minimally improved discrimination (*R*^2^ = 0.222–0.226; Δ = +0.004), and adding the number of vasoactive agents provided only marginal gain (*R*^2^ = 0.233; Δ = +0.011). In contrast, inclusion of VIS produced a substantial improvement in model performance (*R*^2^ = 0.286; Δ = +0.060; likelihood-ratio *P* < 0.001), without significant interaction with SCAI stage, suggesting that the prognostic burden of vasoactive support is consistent across the severity spectrum. Higher VIS was independently associated with mortality [OR = 1.06 per 10-point increase, 95% CI (1.04–1.07); *P* < 0.001]. In sensitivity analyses, the association remained significant in patients with prospectively assigned SCAI stages (OR = 1.04, 95% CI = 1.03–1.07; *P* < 0.001) and in those with retrospectively assigned SCAI stages (OR = 1.07, 95% CI = 1.03–1.10; *P* < 0.001).

Multicollinearity was low (variance inflation factor <2 for all variables), and model discrimination was good (AUC = 0.84; 95% CI = 0.81–0.86).

## Discussion

In this contemporary bicentric cohort of CS patients, managed in an era of multimodal vasoactive support and short-term mechanical circulatory assistance, we identified three main findings. First, substantial heterogeneity in outcomes was observed within each SCAI stage according to baseline vasoactive strategies, suggesting that categorical staging alone does not fully capture prognostic variability. Second, although the number of vasoactive agents was associated with mortality, it proved a crude prognostic marker: increasing the number of agents did not translate into a consistent mortality gradient, whereas vasoactive intensity quantified by VIS was independently and uniformly associated with mortality across all SCAI stages. Third, VIS provided substantial incremental prognostic value beyond both SCAI staging and the number of agents, suggesting that the magnitude of vasoactive support, rather than its categorical composition, better reflects the underlying degree of circulatory failure.

In line with previous registries,^3,4^ mortality increased progressively across SCAI stages, yet substantial heterogeneity persisted within stages, supporting the potential value of complementary prognostic markers within SCAI stages.

This heterogeneity was particularly apparent in our cohort, which reflects contemporary CS management characterized by multimodal vasoactive strategies^12^ including vasopressin analogues,^11^ with no dopamine and limited epinephrine use, a context in which the specific composition and intensity of vasoactive support may carry distinct prognostic implications.

Vasoactive intensity closely paralleled shock severity, with single-agent therapy predominating in stage C, two-drug combinations in stage D, and ≥3-agent regimens mainly in advanced stages. Within-stage analyses revealed that dobutamine alone was associated with lower mortality than norepinephrine alone in stage C, whereas no consistent mortality gradient was observed across strategies in stage D, suggesting that overall haemodynamic severity outweighs specific drug selection in more advanced shock. The association between vasoactive intensity and mortality was consistent across SCAI stages (*P* interaction = 0.43), indicating that escalating support reflects a stage-independent gradient of circulatory failure.

Importantly, while the number of vasoactive agents captured therapeutic escalation, it remained a crude prognostic marker. Current CS guidance relies on SCAI staging and clinical hypoperfusion markers without incorporating a standardized measure of vasoactive intensity.^9^

Although VIS has been associated with outcomes in critically ill patients,^13^ prior studies have shown that baseline or dynamic VIS within the first 24 h may improve risk stratification beyond SCAI staging in large cohorts^14^; however, these analyses have largely focused on selected populations excluding mixed shock—which carries the highest mortality—and post-cardiotomy profiles, and VIS remains rarely integrated into CS risk stratification.

In our study, admission VIS substantially outperformed drug count (Tjur *R*^2^ = 0.286 vs. 0.233) and provided incremental prognostic value beyond SCAI staging, suggesting that systematic VIS assessment may provide incremental prognostic information beyond SCAI staging during the early evaluation of CS.

## Limitations

This study has several limitations. Its retrospective design precludes causal inference, and residual confounding by indication cannot be excluded despite adjustment for major severity markers. Vasoactive strategies reflect early treatment patterns, and subsequent therapeutic modifications were not fully captured. Although all patients were classified using the standardized 2022 SCAI criteria, approximately one-third of the cohort required retrospective SCAI stage assignment based on routinely collected admission clinical, biological, and haemodynamic data. Consequently, some degree of SCAI stage misclassification cannot be excluded, and formal inter-rater reliability was not assessed. Some combinations were represented by small patient numbers, limiting statistical power in within-stage analyses. Although bicentric, no significant centre effect was detected. Finally, both centres are tertiary academic CICUs, and generalizability to community hospitals with different vasoactive practice patterns may be limited, although the inclusion of inter-hospital transfers to expert centres, an integral component of contemporary cardiogenic shock care, supports the representativeness of our cohort, which remains broadly consistent with contemporary CS populations. Finally, VIS was calculated within the first 6 h after CICU admission and should be considered an early severity marker rather than a measure of the entire shock trajectory.

## Conclusion

Taken together, vasoactive intensity quantified by the vasoactive–inotropic score provided substantially better discrimination of 90-day mortality than SCAI stage or the simple count of vasoactive drugs, and fully captured the prognostic information conveyed by the number of agents. These findings suggest that incorporating VIS into early assessment may provide incremental prognostic information beyond SCAI staging.

## Data Availability

The datasets used and/or analysed during the current study are available from the corresponding author on reasonable request.

## AI tools

AI LLM used for English translation and grammar.
